# SEOM–GEICAM–SOLTI clinical guidelines in advanced breast cancer (2022)

**DOI:** 10.1007/s12094-023-03203-8

**Published:** 2023-05-06

**Authors:** Jose Angel Garcia-Saenz, Isabel Blancas, Isabel Echavarria, Carmen Hinojo, Mireia Margeli, Fernando Moreno, Sonia Pernas, Teresa Ramon y Cajal, Nuria Ribelles, Meritxell Bellet

**Affiliations:** 1 Medical Oncology Department, Hospital Clínico San Carlos, Instituto de Investigación Sanitaria San Carlos (IdISSC), CIBERONC, Madrid, Spain; 2 Hospital Universitario San Cecilio, Instituto de Investigación Biosanitaria de Granada (Ibs.Granada) and Medicine Departmen, Granada University, Granada, Spain; 3 Hospital General Universitario Gregorio Marañón, Instituto de Investigación Sanitaria Gregorio Marañon (IiSGM), CIBERONC, Madrid, Spain; 4 Hospital Universitario Marqués de Valdecilla, Santander, Spain; 5 Institut Català d’Oncologia (ICO)-Badalona (Hospital Germans Trias i Pujol), B-ARGO (Badalona Applied Research Group in Oncology) and CARE (Translational Program in Cancer Research), Badalona, Spain; 6 Institut Català d’Oncologia (ICO)-L’Hospitalet, Institut d’Investigacio Biomedica de Bellvitge (IDIBELL), L’Hospitalet de Llobregat, Barcelona, Spain; 7 Hospital de la Santa Creu i Sant Pau, Barcelona, Spain; 8 UGCI Oncología Intercentros, Hospitales Universitarios Regional y Virgen de la Victoria (IBIMA), Málaga, Spain; 9 Hospital Universitario Vall D’Hebron, and Vall d’Hebron Institute of Oncology (VHIO), Barcelona, Spain

**Keywords:** Advanced breast cancer, ABC, Guidelines, Endocrine therapy, Anti-HER2 therapy, IOT, ADC

## Abstract

Advanced breast cancer represents a challenge for patients and for physicians due its dynamic genomic changes yielding to a resistance to treatments. The main goal is to improve quality of live and survival of the patients through the most appropriate subsequent therapies based on the knowledge of the natural history of the disease. In these guidelines, we summarize current evidence and available therapies for the medical management of advanced breast cancer.

## Introduction

Breast cancer is a major public health problem, because of both its high incidence and prevalence, as well as its morbidity and mortality rate [1]. Although breast cancer is one of the most treatment sensitive solid tumors, advanced breast cancer (ABC) is not curable in most cases. Therefore, therapy is usually palliative at this stage of the disease and the main aims are to improve the patient’s quality of life and to prolong their survival.

A better understanding of the tumor biology has allowed to search for more specific and well-tolerated specific therapies. Novel endocrine and cytotoxic agents, the emerging of molecular targeted drugs, together with the different therapeutic sequences administered throughout the disease have modified the natural history of ABC. The aim of this document is to summarize current evidence and to give evidence-based recommendations for clinical practice. As regulatory status for new drugs are dynamic, all therapies for which robust evidence of activity is shown in clinical trials are included in the body of the manuscript, regardless of Spanish Health Agency approval status at the time of this guideline publication. However, this current status is mentioned within the algorithms presented.

## Methodology

These SEOM Guidelines have been developed with the consensus of ten breast cancer medical oncologists from the cooperative groups GEICAM (Spanish Breast Cancer Research Group) and SOLTI (Spanish Collaborative Group for the Study, Treatment and Other Experimental Strategies in Solid Tumors). To assign a level of evidence and a grade of recommendation to the different statements of this treatment guideline, it was decided to use the Infectious Diseases Society of America-US Public Health Service Grading System for Ranking Recommendations in Clinical Guidelines to determine the quality of evidence and strength of recommendation in each of the consensus recommendations (Table 1).

**Table 1:** Strength of recommendation and quality of evidence score

Category, grade	Definition
*Strength of recommendation*	
A	Good evidence to support a recommendation for use
B	Moderate evidence to support a recommendation for use
C	Poor evidence to support a recommendation
D	Moderate evidence to support a recommendation against use
E	Good evidence to support a recommendation against use
*Quality of evidence*	
I	Evidence from ≥ 1 properly randomized, controlled trial
II	Evidence from ≥ 1 well-designed clinical trial, without randomization; from cohort or case-controlled analytic studies (preferably from > 1 center); from multiple time series; or from dramatic results from uncontrolled experiments
III	Evidence from opinions of respected authorities, based on clinical experience, descriptive studies, or reports of expert committees

## Pathology and molecular biology

At the time of first diagnosis of ABC (newly diagnoses or relapsed), a tumor biopsy is necessary to determine estrogen receptor (ER), progesterone receptor (PR), and human epidermal growth factor receptor 2 (HER2) status [I, A] [2].

HER2-low breast cancers (defined as immunohistochemically 1+ or 2+ and lack of HER2 gene amplification measured by in situ hybridization) should be identified, as they may derive benefit from targeted therapies as explained below.

In patients with metastatic triple negative breast cancer (TNBC), programmed death-ligand 1 (PD-L1) status should be determined by immunohistochemistry, to decide if therapy with immune checkpoint inhibitors should be incorporated to first-line treatment [I, A].

In HER2-negative MBC, germline BRCA1/2 mutations (gBRCAm) status should be tested since treatment with PARP inhibitors could be indicated [I, A]. Somatic sequencing [II-A] cannot fully substitute germline BRCA testing but could guide to confirm a potential gBRCAm status.

In ER and/or PR positive HER2-negative ABC patients, phosphatidylinositol-4,5-bisphosphate 3-kinase catalytic subunit alpha (PIK3CA) mutations should be assessed [I-A] to consider the use of PIK3CA inhibitors [3].

Microsatellite instability (MSI-high), an infrequent biomarker in breast cancer (1.7%) [I, C], could be considered if therapy with pembrolizumab is available. Other infrequent biomarkers in breast cancer (< 0.1%) that might also be tested, are the NTRK fusions/translocations, since their detection are associated to a high efficacy of NKTR inhibitors irrespectively of the type of primary tumor (agnostic indication) [I, C].

According to the ESCAT guidelines [4] that include high number of level II alterations, tumor multigene next-generation sequencing (NGS) and circulating tumor DNA (ctDNA) genomic profiling tests are still not routinely recommended, although they should be offered to MBC patients if it may change treatment or enable their inclusion in clinical trials [V, B].

**Fig. 1:**
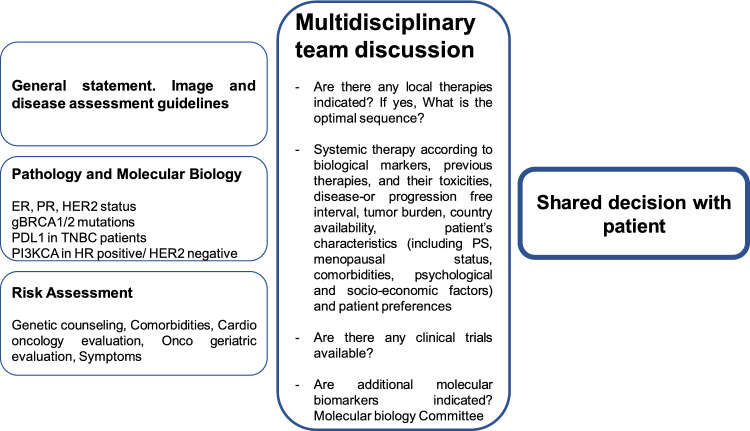
Algorithm proposal for decision-making on the treatment of patients with ABC

Figure 1 briefly summarizes the different variables to be considered for decision-making on the treatment of ABC patients.

## General statement: image and disease assessment guidelines

Systemic therapy is the standard-of-care for ABC, but locoregional therapies may be included if clinically indicated for an optimal management; furthermore, referral to the supportive care team should be also considered as soon as possible. Moreover, in elderly patients a comprehensive geriatric assessment is highly recommended. Incurability and treatment goals should be discussed. Genetic counseling and cardio-oncology evaluation should also be considered as part of the multidisciplinary approach for patients with ABC.

Biopsy of novel metastatic disease or recurrence should be performed whenever possible, since discordances in ER, PR and HER2-status between primary and metastatic tumor may occur [I, B]. In case of discordances, there are no specific recommendations on which of the results should be taken into account, but the use of endocrine therapy or anti-HER2 therapy should be considered if there has been an ER/PR or HER2-positive biopsy at any time [III, C].

Staging of metastatic disease should include thoracic and abdominal computed tomography (CT) and bone scan [II, A]. (^18^F-FDG) PET-CT may be used instead of CT and bone scan [II, B]. PET-CT may be used to confirm oligometastatic disease in which local approaches might be considered. However, PET-CT is not recommended to assess treatment response and initiate a new therapeutic line, since a greater advantage of PET-CT compared to CT or bone scan in this issue has not been established [5]. It should not be forgotten that clinical trials use CT and bone scan to establish the status of progression disease. Although tumor marker assessment may be useful, changes in treatment lines should not be performed based on their sole increase, in the absence of documented disease progression [II, C].

Central nervous system (CNS) screening in asymptomatic patients is not routinely recommended, although it may be considered in patients at a higher risk (TNBC and HER2-positive) if the detection of CNS metastases would change the treatment choice [V, C].

## HR-positive/HER2-negative ABC (Fig. 2)

**Fig. 2:**
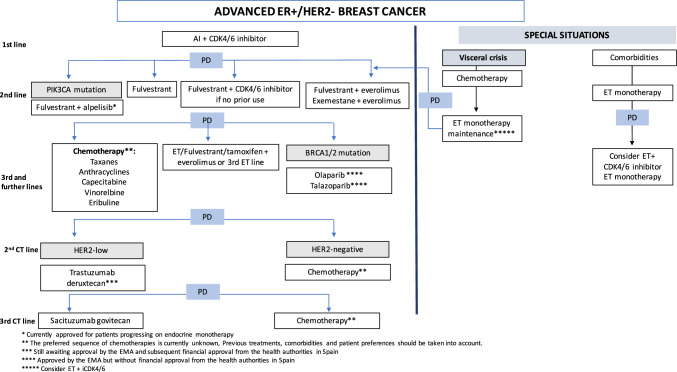
Algorithm advanced ER+/HER2- breast cancer

### General statements for HR+/HER2- ABC

Sequential endocrine therapies (ETs), in monotherapy or combined with targeted therapies are the preferred choices as initial treatment for HR+/HER2-negative ABC [I, A]. Front-line chemotherapy should only be considered for visceral crisis, defined as a life-threatening disease a risk of imminent organ failure and for which a rapid response is needed. High tumor burden or visceral disease is not synonymous with visceral crisis and thus, does not imply the need for chemotherapy. Pre- and perimenopausal women should undergo ovarian function suppression or ablation and be treated as postmenopausal women [I, A].

First-line treatment (see below) is considered for patients with no prior endocrine therapy (ET) or for those who progress at least 12 months after completing adjuvant treatment (endocrine-sensitive scenario). However, endocrine resistance inevitable occurs. Primary endocrine resistance refers to patients recurring within the first 2 years of adjuvant ET or progressing in the first 6 months of ET in the advanced setting. Secondary resistance has been defined as disease recurrence after at least 2 years of adjuvant ET and, within a year from its completion, or those progressing after at least 6 months of ET in the advanced setting.

Concomitant chemotherapy and endocrine therapy is not recommended [II, D].

### First-line treatment

ET in combination with CDK4/6 inhibitors (CDK4/6i) has become the standard-of-care for first- or second-line treatment of ER-positive/HER2-negative ABC, due to their efficacy results, toxicity profile and quality of life data [I, A].

All the three CDK4/6i approved (palbociclib, ribociclib and abemaciclib) have demonstrated an improvement in progression free survival (PFS) ranging from 9 to 14 months as first-line treatments and are suitable options in this setting. Ribociclib has demonstrated a significant improvement in overall survival (OS) in the first-line setting both in the MONALEESA-2, 3 and 7 trials: MONALEESA-7 was conducted exclusively in premenopausal women, while MONALEESA-3 also includes hormone-resistant patients. Abemaciclib has demonstrated an OS benefit in hormone-refractory patients in the MONARCH-2 trial, whereas final mature data in the first-line setting from the MONARCH-3 trial is still awaited. Palbociclib has failed to show a significant improvement in OS in the first-line and have a marginal benefit in second/third-line setting which included patients treated with prior chemotherapy. No randomized study has compared the different CDK4/6i, so indirect comparisons across trials should be cautiously considered, particularly because populations are not completely interchangeable. Although no trial has assessed the preference of first vs second-line CDK4/6i treatment, the general recommendation is to use them as soon as possible [II, C] [6, 7].

In the first-line setting, CDK4/6i are usually combined with aromatase inhibitors (AI), although the MONALEESA-3 trial, with fulvestrant and ribociclib, included around half of the patients in first-line, and MONARCH-2 trial, with fulvestrant and abemaciclib also included patients in first-line. Therefore, ribociclib with fulvestrant or abemaciclib with fulvestrant may be an adequate option for first-line treatment (I, A). The benefit of fulvestrant combined with CDK4/6i compared to AI was assessed in combination with palbociclib in the PARSIFAL trial, in which fulvestrant–palbociclib failed to be superior, and also to be non-inferior in PFS compared to letrozole–palbociclib [II, C] [8]. ET should be reserved to patients with comorbidities preventing the use of an iCDK4/6. In this case, both AI and fulvestrant are suitable options, although the FALCON trial demonstrated a benefit in favor of fulvestrant in the first-line setting in endocrine-naive patients, driven by those without visceral disease [I, A]. There is scarce evidence for maintenance therapy with CDK4/6i after chemotherapy, and thus, in general, it should be carried out with endocrine monotherapy. Nevertheless, in the MONALEESA-7 and CompLEEment-1 trials, there were a proportion of patients receiving maintenance therapy with ET + iCDK4/6 after initial chemotherapy in the advanced setting. Hence, it seems reasonable to consider this combination after clinical stabilization or chemotherapy discontinuation due to toxicity for the few cases (e.g., visceral crisis) in which chemotherapy were delivered first (III, D).

### Second-line treatment

In patients progressing on AI or within the first year after completion of the adjuvant ET, treatment with CDK4/6i and fulvestrant is recommended [I, A] [9].

PIK3CA mutations occur in approximately 40% of ABC. Alpelisib in combination with fulvestrant demonstrated an improvement in PFS in patients with PIK3CA mutations (11.0 vs 5.5 months) and a non-significant benefit in OS (39.3 vs. 31.4 months) (3). Only a small proportion of patients included in the SOLAR-1 trial had received iCDK4/6; however, the phase 2 BYLieve trial has evaluated alpelisib after iCDK4/6 in combination with AI or fulvestrant observing a median PFS of 5.6 and 7.3 months, respectively. Alpelisib is now approved by the EMA for patients progressing on endocrine monotherapy, although in other countries it is approved irrespectively of prior iCDK4/6 treatment [I, B] [10, 11].

Everolimus in combination with AI or fulvestrant is also an option for second-line treatment [I, B], with a PFS benefit of 6.9 vs 2.8 months with exemestane and everolimus, and 10.3 *vs.* 5.1 months with fulvestrant and everolimus over fulvestrant monotherapy. However, this latter PFS benefit could be overestimated due to a high level of informative censoring [I, B] [12]. Of note, these two combinations were tested before the use of iCDK4/6, so their efficacy data after iCDK4/6 are scarce.

### Chemotherapy

Chemotherapy is still a mainstay in the treatment of advanced HR+/HER2- breast cancer [I, A]. No specific algorithm for chemotherapeutic agents is available, and election of the chemotherapy regimen will be based on the patient characteristics, previous treatments, expected toxicities and patient’s preferences. Alopecia is often a significant concern for patients that should be considered.

Although both combination or monotherapies are adequate options, generally sequential monotherapies are preferred. Nevertheless in selected cases, with high tumor burden or in which a rapid response is needed combinations may be indicated. Taxanes and anthracyclines should be considered in patients who have not received them in the neo/adjuvant setting. Re-use of taxanes is feasible, especially if more than a year has elapsed since its previous use. As for anthracyclines, although re-use can be considered, maximum cumulative doses should not be exceeded, and liposomal formulations are preferred as their associated with less cardiotoxicity.

Capecitabine is an option frequently used in early ABC treatment lines due to its oral availability and favorable toxicity profile; before treatment starting, and to avoid severe, even lethal toxicities an assessment of a potential DPD deficiency by genotyping DPYD gene polymorphisms is recommended [13].

Other chemotherapy agents useful in this metastatic setting are eribulin, vinorelbine, gemcitabine, carboplatin, and, more rarely, old regimens such as CMF (cyclophosphamide, methotrexate, and 5-fluorouracil) in classical iv or oral metronomic versions. In this setting, eribulin has shown to provide a benefit in overall survival [14].

### Bevacizumab

Treatment with bevacizumab plus chemotherapy as the first chemotherapy line may be considered in selected patients due to PFS benefit, although it has not demonstrated a significant benefit in OS [I, C] [15].

### Antibody–drug conjugates (ADC)

Results from a study comparing trastuzumab deruxtecan (T-Dxd) vs. therapy per physician’s choice (TPC) in paHER2-low ABCtients with HER2-low tumors, mainly hormone receptor positive, were recently published [16] (see “”). A significant improvement in PFS and OS was found. Treatment was delivered as a second-line chemotherapy in most cases. In addition, a second phase 3 trial comparing sacituzumab govitecan vs. TPC in HR+ HER2- population as a late-line treatment (3 median prior chemotherapy lines) showed a significant improvement in PFS and OS [17] although the absolute magnitude of benefit was smaller than in the counterpart trial for TNBC patients.

### PARP inhibitors (PARPi)

Both olaparib and talazoparib have shown superiority in terms of PFS to single-agent chemotherapy at physician’s choice in two phase 3 studies including patients harboring gBRCAm with tumors HER2-negative patients. Benefit was observed irrespective of hormonal receptor status [18]. Therefore, in the HR+ setting, for this specific population treatment with PARPi should be considered after first-line ET + CDK4/6i [I, C].

## HR-negative/HER2-negative ABC

**Fig. 3:**
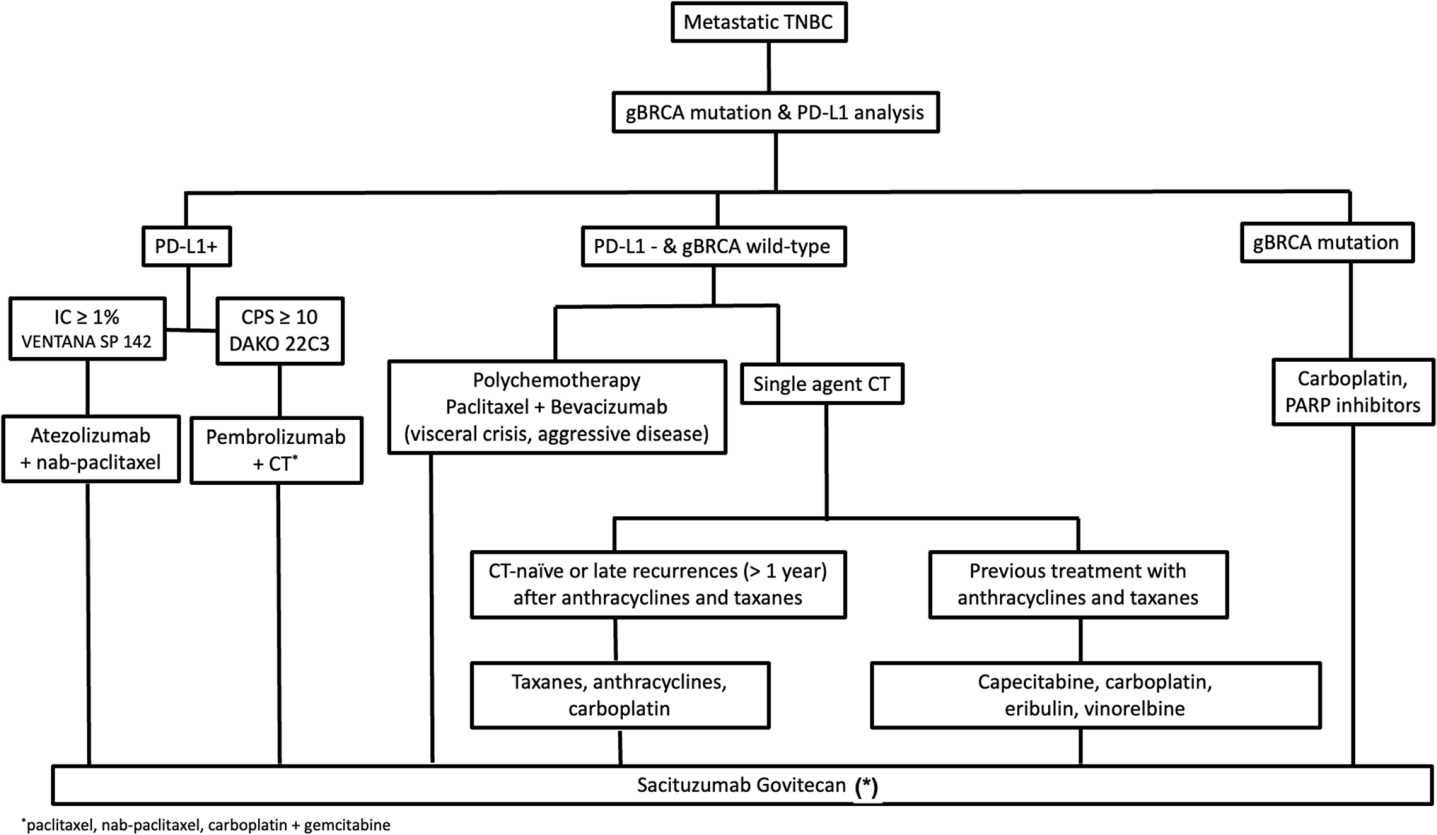
Algorithm advanced TNBC. (*) T-Dxd could be considered if patient is not suitable for sacituzumab govitecan. sacituzumab govitecan) is also preferred as second line if patient has received a previous neo/adjuvant chemotherapy schedule

TNBC is clinically defined by the lack of the ER, PR and HER2 expression. TNBC accounts for nearly 15% of all breast cancers and is associated with worse prognosis when compared to other subtypes (Fig. 3).

### Biomarker analysis in metastatic TNBC

TNBC is a heterogeneous disease and recent advances in genomics and molecular profiling have helped better definition of TNBC subtypes with distinct biologic drivers to guide the therapeutic decisions [19]. Patients with PD-L1-positive tumors may benefit more from checkpoint inhibitor-based combinations and, therefore, PD-L1 should be tested before first-line treatment for ABC [I, A]. Patients with metastatic TNBC who may be considered for treatment with PARPi should undergo testing for germline BRCA1 and BRCA2 pathogenic or likely pathogenic mutations [I, B]. Other biomarkers such as tumor mutational burden, deficient mismatch repair/microsatellite instability-high and NTRK fusions could be tested in patients candidates to pembrolizumab or TRK inhibitors, respectively [III, B] [20].

### First-line treatment

The choice of first-line treatment should be guided by the expression of PD-L1 and gBRCAm analysis.

#### PD-L1-positive metastatic TNBC

The addition of a checkpoint inhibitor to a standard chemotherapy has been evaluated in three phase III clinical trials. The IMpassion130 trial randomized patients with advanced TNBC to receive nab-paclitaxel with either atezolizumab or placebo. PD-L1 IC status was assessed with the Ventana SP142 assay (positivity defined as ≥ 1%). PFS was significantly longer with atezolizumab in both the intention to treat (ITT) population (7.2 vs 5.5 months) and the PD-L1+ subgroup (7.5 vs 5.0 months) [21]. The OS was not statistically better with the addition of atezolizumab in the ITT population, though a clinically meaningful 7-month improvement in median OS was seen with atezolizumab in the PD-L1+ subgroup (25 vs 18 months), but formal significance testing was not performed due to the prespecified hierarchical statistical analysis plan [22]. The similarly designed IMpassion131 trial explores first-line paclitaxel with or without atezolizumab in advanced TNBC. The primary endpoint of PFS was not met in either the PD-L1+ subgroup or the ITT population [23].

KEYNOTE-355 trial randomized patients with metastatic TNBC to TPC (nab-paclitaxel, paclitaxel, gemcitabine/carboplatin) plus pembrolizumab or placebo. Administration of pembrolizumab was associated with an improvement in PFS in patients with combined positive score (CPS) ≥ 10 PD-L1-positive metastatic TNBC (9.7 vs 5.6 months) but not for patients with lower levels of PD-L1 expression [24]. OS was also statistically prolonged in the CPS ≥ 10 population (23 vs 15.1 months) [25].

Given these results, atezolizumab plus nab-paclitaxel and pembrolizumab plus chemotherapy (several regimens) are the preferred options for metastatic TNBC patients with PD-L1 expression in ≥ 1% of tumor infiltrating immune cells determined with SP142 assay and with CPS ≥ 10 by 22C3 assay, respectively [Atezolizumab II, A; Pembrolizumab I, A].

### PD-L1-negative gBRCAm metastatic TNBC (see genetic testing and BRCA-associated disease)

#### PD-L1-negative gBRCA wild-type metastatic TNBC

The choice between different cytotoxic agents is conditioned by several factors (previous exposure, disease-free interval, comorbidities) and must be considered individually. Although polychemotherapy provides higher rates of objective response and longer time to progression, it is associated with higher toxicity and the OS benefit is small [26]. Therefore, sequential use of single-agent chemotherapy is preferred, hence combination therapies should be limited for patients with aggressive, symptomatic, or life-threatening disease [I, A]. Longer chemotherapy duration is associated with higher efficacy as well as increased risk of toxicity [27]. Thereby, except for conventional anthracyclines, chemotherapy should be administered until disease progression or unacceptable toxicity [I, B].

In CT-naïve patients, taxanes or anthracyclines are the preferred first-line treatment [I, A]. This recommendation is also valid for patients with late recurrences (> 1 year) after completing neo/adjuvant anthracyclines and/or taxanes [I, A]. TNT trial showed similar efficacy of carboplatin compared with docetaxel and should be also considered an option irrespective of gBRCAm status and previous exposure to anthracyclines and/or taxanes [I, A] [28]. Bevacizumab improves PFS and ORR but not OS when combined with taxanes or capecitabine in HER2-negative ABC patients and may be considered for patients with visceral crisis or high symptomatic disease [I, C] [29]. Maintenance therapy with bevacizumab plus capecitabine (compared to bevacizumab alone) after induction first-line treatment with bevacizumab plus docetaxel improves PFS and OS for HER2-negative ABC patients and may be considered for selected cases [I, C] [30].

### Second and subsequent lines of treatment

After progression to first-line chemotherapy, anthracyclines, if not previously given, are an option for patients who received taxanes and vice versa. Capecitabine, eribulin, carboplatin, gemcitabine and vinorelbine are active agents as single agents in patients pretreated with anthracyclines and taxanes [I, A] [31–34]. Due to the lack of high-quality comparative data, the most efficacious sequencing of chemotherapy agents in the treatment of ABC has yet to be defined. Treatment decision should be individualized considering different toxicity profiles, previous exposure, and patient preferences. In case of considering capecitabine or other fluoropyrimidines, it is highly recommended to genotype DPYD polymorphisms [13].

Sacituzumab govitecan (SG) is an antibody–drug conjugate (ADC) composed of a Trop-2 antibody coupled to an SN-38 payload, via a proprietary, hydrolyzable linker. In the phase III trial ASCENT study, SG was compared with single-agent chemotherapy of the TPC (eribulin, vinorelbine, gemcitabine and capecitabine) in TNBC patients with relapsed or refractory metastatic disease treated with two or more previous standard chemotherapy regimens (at least one of them in the metastatic setting). SG showed a significant improvement in median PFS (5.6 vs 1.7 months) and median OS (12.1 vs 6.7 months), compared with TPC [35]. Based on these results, SG should be considered as the preferred treatment after anthracyclines and taxanes [I, A]. More recently, another phase 3 trial (DESTINY-04) tested trastuzumab–deruxtecan (T-Dxd) vs. TPC (taxanes, eribulin, capecitabine, gemcitabine) in HER2-low patients as a first- or second-line chemotherapy for the advanced setting (see “HER2-low ABC”), The study showed a benefit in terms of PFS and OS in the small group of TN patients included (*N* = 60). Therefore, although with less evidence, treatment with T-Dxd may also be an option, in particular when safety profiles (diarrhea and neutropenia for SG, emesis and pneumonitis for T-Dxd) are taken into account [I, B] [16].

## HER2-positive ABC (Fig. 4)

**Fig. 4:**
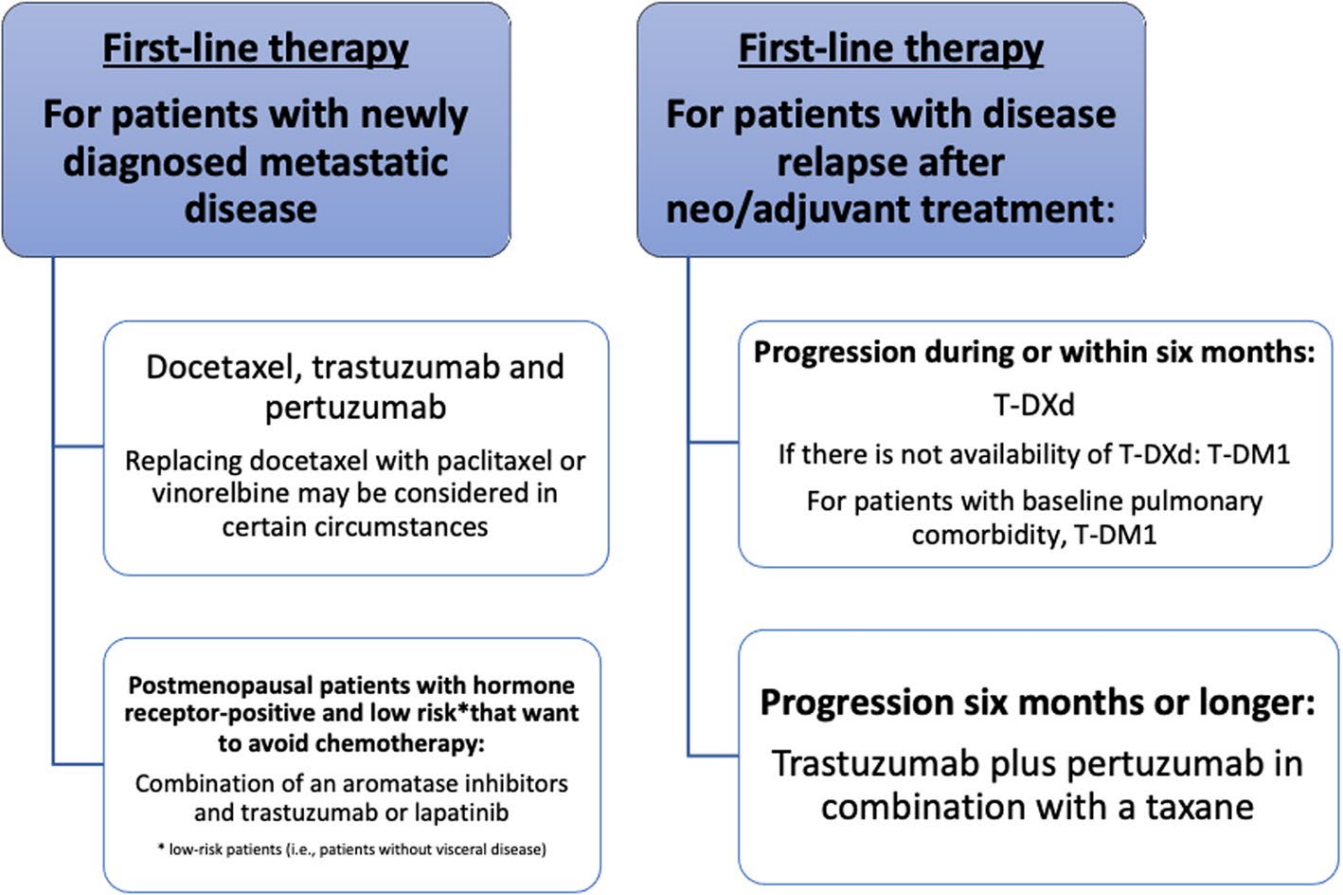
Algorithm for first-line advanced HER2+ breast cancer

A high level of HER2 overexpression, as determined by either 3+ staining by immunohistochemistry (IHC) for the HER2 protein or evidence of *HER2* gene amplification by fluorescence in situ hybridization (FISH ratio ≥ 2.0 or HER2 copy number ≥ 6.0), is a strong predictive factor for sensitivity to HER2-targeted agents, and these criteria should be used to select patients for these drugs. For patients with metastatic HER2-positive breast cancer, anti-HER2-directed therapy should be included in the treatment regimen [I, A] [36].

### First-line therapy

#### For patients with newly diagnosed metastatic disease

First-line treatment with trastuzumab and pertuzumab in combination with taxanes is associated with improvement in PFS, ORR and OS versus chemotherapy alone (CLEOPATRA trial) [I, A] [37]. Vinorelbine instead of taxanes may be considered in certain circumstances [III, C] [38].

In postmenopausal patients with hormone HR(+) and HER2-positive tumors, the combination of aromatase inhibitors and an anti-HER2 agent (trastuzumab or lapatinib) has shown an increase in PFS, ORR but not OS versus endocrine therapy alone [39–41]. Overall, the efficacy with these combinations seems inferior to that reached with chemotherapy plus anti-HER therapy, and should be limited to low-risk or unfit patients [II, B].

### For patients with disease relapse after neo/adjuvant treatment

- For patients who progress during or within 6 months of adjuvant treatment:
  In patients with relapse after adjuvant trastuzumab, there is limited scientific evidence on the best treatment option, since few patients with these characteristics were included in the pivotal CLEOPATRA trial. T-DM1 was superior to lapatinib plus capecitabine in terms of response rate, PFS and OS in patients pretreated with either first-line trastuzumab combinations or early relapses after trastuzumab adjuvant therapy [42].
  In the phase III Destiny-Breast03 trial including patients with progression on a trastuzumab- and taxane-containing regimen, median PFS and OS was significantly superior for T-DXd vs T-DM1 and the benefit was observed in all the sub-groups analyzed [43]. For patients who progress during or within 6 months of adjuvant treatment, T-DXd is the preferred option for this population [I, A].
- For patients who progress 6 months or longer after the completion of adjuvant therapy:
  The most accepted recommendation is trastuzumab plus pertuzumab in combination with a taxane rather than other agents.

### Second-line therapy

Several studies have shown that there is a benefit in continuing with second-line anti-HER2 therapy, after progression during or following first-line treatment with trastuzumab [I, A]. Based on Destiny-Breast03 trial for patients who experience disease progression following a trastuzumab-containing regimen, T-DXd is the preferred second-line option for this population ([I, A] [43].

### Third-line and further therapy

Patients with advanced HER2-positive breast cancer, who have been treated with two or more lines of anti-HER2 therapy, may benefit from a third or further line of anti-HER2 [I, A]. The choice is often based on prior treatments, patient preference, prior toxicities, and drug availability. In a randomized trial including heavily pretreated patients (median of four prior lines of therapy, but no prior T-Dxd), PFS and OS rates were superior in patients receiving tucatinib–capecitabine–trastuzumab compared to those receiving capecitabine–trastuzumab only [44]. Therefore, tucatinib–trastuzumab–capecitabine may be a good option for patients treated with previous T-DM1 and/or T-Dxd [II, B].

The combination of lapatinib plus trastuzumab in patients progressing on trastuzumab showed a higher PFS and OS versus lapatinib alone. The benefit was more notable in the subgroup of HR-negative patients [II, B] [45].

For patients who experience disease progression following a trastuzumab-containing regimen in the metastatic setting, options include T-DXd (preferred regimen), TDM-1, continuation of trastuzumab with a different chemotherapy partner, tucatinib–capecitabine–trastuzumab or a tyrosine kinase-based combination, and anti-HER2 agents plus endocrine therapy in ER-positive/HER2-positive.

The optimal number of lines of anti-HER2 therapy for metastatic breast cancer is currently unknown, although available data suggest benefits are maintained in third-line and beyond [II, B].

## Genetic testing and BRCA-associated disease

*BRCA and BRCA2 are* high-penetrance cancer susceptibility genes involved in homologous recombination DNA repair that are mutated in 5% of unselected BC patients [46]. In addition to cancer family history and single personal criteria related to early onset or phenotype, testing criteria for *BRCA1* and *BRCA2* genes have been expanded over time to optimize identification of putative patients who might benefit from active therapies that create DNA double-strand breaks or stalled replication forks, such as the platinum salts or PARPi. Therefore, updated germline [I, A] and somatic [II, A] testing indications include patients with metastatic HER2-negative disease who had previously been treated with an anthracycline and a taxane in the (neo)adjuvant or metastatic setting. Those patients with HR-positive BC should have progressed on or after prior endocrine therapy or be considered unsuitable for endocrine therapy. Such clinical criteria are established regardless of family history, due to the possibility of lacking BC-affected relatives in 20–70% of cases.

In platinum-naïve patients with gBRCAm TNBC, platinum chemotherapy should be preferred to taxanes based on the results of one phase III trial comparing first-line carboplatin to docetaxel. Unlike the unselected population in which no difference between arms was observed, gBRCAm carriers had improved ORR (68% vs 33%, *p* = 0.03) and median PFS (6.8 vs 4.8 months) with carboplatin. However, not benefit in OS was seen [I, A] [28]. In addition, two PARPis have been evaluated in two phase III trials, OlympiaD and EMBRACA in which patients were randomized to olaparib or talazoparib, respectively, vs TPC. In both studies, patients who received olaparib or talazoparib had longer PFS (HR 0.58 and HR 0.54, respectively), higher ORR and better quality of life in first-to-third-lines than those receiving single-agent therapy of the physician’s choice [I, A]. These trials failed to show a statistically improvement in OS [18, 47, 50, 51].

Patients with HR-positive gBRCAm advanced disease should receive first-line endocrine therapy with CDK4/6 inhibitors and consider PARPi beyond progression [I, A].

## HER2-low ABC

More than half of breast cancers historically categorized as HER2-negative express low levels of HER2 (ERBB2), defined as immunohistochemically (IHC) 1+ or 2+ and lack of HER2 gene amplification measured by in situ hybridization. These “HER2-low” tumors are, however, a heterogeneous population including both HR-positive and HR-negative breast cancers that may vary in prognosis and response to systemic treatments.

T-DXd has shown significant improvements in PFS and OS compared to the physician’s choice of chemotherapy among patients with HER2-low metastatic breast cancer, regardless of HR status, in one study including nearly 90% of HR-positive tumors [I, A] [16]. This highlights the clinical relevance of the HER2-low patient population and supports the importance to implement reproducible and sensitive assays to measure HER2-low expression, as the reproducibility of HER2-low among pathologists may be suboptimal.

## Management and treatment of CNS metastases

The incidence of brain metastases in ABC has increased in recent decades, mainly because of the improved survival of these patients. Approximately, more than one-third of HER2+ BC patients, one-third of mTNBC, and 15% of HR+ HER2- ABC patients will develop brain metastases [48]. Progression in the CNS is still a therapeutic challenge due to the negative impact in quality of life and survival [49].

Early detection by MRI and treatment of CNS may improve quality of life, management, and perhaps survival in women with metastatic BC, especially HER2-positive or TN subtypes. Several clinical trials are being carried out to address this issue [50].

The landscape of the treatment of brain metastases in breast cancer has changed in the last years with an increasing use of systemic therapy and focal stereotactic radiosurgery to the detriment of surgery and whole brain radiotherapy. In all patients, a multidisciplinary approach is essential for optimal management.

### Local therapy

*Surgical resection*: of single brain metastases in patients with controlled systemic disease remains the first option [II-A] [51].

*Stereotactic radiosurgery*: is generally preferred for patients with oligometastatic disease and for lesions not surgically accessible [I, A]. It may also be considered for patients with a higher number of brain metastasis after complete resection [II, A]. SRS has become an important strategy to improve local control with few side effects.

*Whole-brain radiation therapy*: it may be considered for patients with numerous brain metastases and poor performance status [II, B]; hippocampus avoidance are strategies to decrease neurocognitive impairment that should be considered.

#### Systemic therapy Her2 positive

A number of anti-HER2 agents have shown intracranial activity even in heavily pretreated patients [52].

- New brain metastases with controlled systemic disease: considered local therapy with the same anti-HER2 regimen [II, B].
- New brain metastases with progressive systemic disease: considered HER2-targeted therapy according to the algorithms for treatment of HER2-positive MBC. Tucatinib/capecitabine/trastuzumab is the preferred regimen, particularly among oligosymptomatic and limited disease, and for patients who prefer to defer radiation [II, B]. T-DXd may also considered, particularly if systemic progression is a clinical issue [II, C].

#### Systemic therapy HER2 negative

- Classical chemotherapy agents such as capecitabine, methotrexate, carboplatin, etoposide, vinorelbine and gemcitabine, have been used in this scenario with limited activity [II, B] [53].
- Bevacizumab can improve CNS response and may be an option particularly in TNBC [III, C] [54].
- The CDK4/6 inhibitors abemaciclib and ribociclib penetrate the blood–brain barrier, and have shown intracranial activity in small early trials [II, C].
- New agents including immune checkpoint inhibitors, PARPi, and drug conjugates are being evaluated in clinical trials for potential intracranial efficacy.

## Oligometastatic breast cancer

Oligometastatic breast cancer, generally defined as low-volume metastatic disease with limited number and size of metastatic lesions (≤ 5 lesions and not necessarily in the same organ) represents up to 10% of the patients with advanced breast cancer. This distinction between oligo- and widely metastatic disease is increasingly recognized because of treatment and survival implications [55].

The standard-of-care for oligometastatic disease in breast cancer is the use of systemic therapy, but given the likelihood of limited tumor burden, it may benefit from a radical approach with the addition of locoregional treatments to all sites of the disease for long-term disease control and eventually cure. Local treatment strategies include surgical excision, radiofrequency, and stereotactic body radiotherapy (SBRT). A meta-analysis of oligometastatic breast cancer patients treated by SBRT offered local control of 97% at 1 year and 90% at 2 years. However, the analysis and comparison of local control at the treated bone lesion between different series are complicated given the heterogeneity of populations and treatments [56].

The optimal management of oligometastatic breast cancer remains unclear due to the scarce and heterogeneity of the existing data. However, selected patients with oligometastatic disease may be offered a multimodal approach with curative intent, including local therapy to all known metastases, if it can be safely accomplished and a multidisciplinary team is involved.

## Male BC

MBC accounts for about 1% of all BC. However, its incidence can be higher among males with either genetic disorders or germline mutations in cancer susceptibility genes (*BRCA2, CHEK2, ATM and PALB2*) regardless of family history [57]. While most males are diagnosed at early stages, diagnostic delays explain a higher rate of de novo metastatic disease (32%) in comparison with female population. In addition, MBC patients have a worse overall prognosis than female BC counterparties.

Management recommendations are mostly extrapolated from either retrospective studies or clinical trials including large number of females. Since luminal B-like/HER2-negative invasive ductal carcinoma is the most frequent subtype occurring in males, both aromatase inhibitors (concurrently to GNRH analogs or orchidectomy) or fulvestrant should be recommended as front-line unless in case of visceral crisis or rapidly progressive disease [III, B]. Recent real-world data studies have suggested similar PFS benefit and favorable safety profile of CDK4/6 inhibitors combined with aromatase inhibitors or fulvestrant that in females [58]. While prospective international collections or clinical trials including newly diagnosed male cases provide some data, it is reasonable to follow similar indications for the management of other subtypes or late lines of advanced disease with PIK3CA or mTOR inhibitors, HER2-targeted therapy, immunotherapy and PARP inhibitors as in females [III, B] [59].

## Supportive and palliative care

ABC is an incurable disease, with a median overall survival ranging 1.5 to more than 5 years, depending on the subtype. In addition to receiving the best antineoplastic treatment along with the appropriate measures to avoid therapy-associated toxicity, patients should be offered optimal symptom control, psychological, social, and spiritual support. Regarding symptom control, antiresorptive agents (zoledronic acid or denosumab) should be considered in the presence of bone metastases to prevent skeletal-related events and as co-adjuvant drugs for pain control. Optimization of oral care should be implemented to minimize the risk of osteonecrosis of the jaw.

Palliative care (PC) is an approach that improves the QoL of patients and their caregivers when facing the problems associated with BC through prevention, early identification and treatment of physical, psychosocial, and spiritual issues. PC should start early in the course of disease, while patients are receiving curative treatment and multidisciplinary and collaborative approaches should be integrated in PC of BC patients. When patients are followed up concurrently by PC and Medical oncologist, they reported better QoL and less depression, received less chemotherapy, and achieved longer overall survival than those who underwent a traditional care model [60].

## Future directions

Currently, several questions in ABC remain unanswered. This is the case, for example, of NGS panel tests or liquid biopsy, which have shown clinical utility for the detection of actionable mutations (*PIK3CA* mutations, *ERBB2* amplification, *gBRCAm*) [20] but are not yet integrated in our routine practice or are on hold until full approval/reimbursement of the therapies for which such molecular alterations apply. In addition, the development of accurate predictive biomarkers of benefit with currently available or future treatments is mandatory, as well as the best technique to determine them.

Regarding novel treatments for ER-positive/HER2-negative patients, several endocrine therapies are in different stages of development, especially for patients with mutations in *ESR1*, acting in different ways on the estrogen receptor (novel oral SERD, PROTAC, etc.). Other agents designed to overcome endocrine resistance are also being developed (AKT inhibitors, fibroblast growth factor inhibitors, and aurora kinase A inhibitors). For ER-negative/HER2-negative MBC, different strategies are being investigated, such as PARP inhibitors, AKT inhibitors, MEK inhibitors or combining check point inhibitors with other immunotherapy or targeted agents. In the development of anti-HER2 therapy, efforts are focused on novel TKIs, antibody–drug conjugates, bispecific antibodies, immunotherapy and agents that inhibit HER2 protein production or induce its destruction. It is also necessary to establish the optimal sequencing of all these therapies and if the combination of some of these agents could improve the results in ABC patients.

## Data Availability

I hereby state that all the Data provided within these Guidelines are fully checked and available. Dr. Jose Angel Garcia-Saenz.
