# Correction to: SEOM–GEICAM–SOLTI clinical guidelines in advanced breast cancer (2022)

**DOI:** 10.1007/s12094-023-03229-y

**Published:** 2023-06-05

**Authors:** Jose Angel Garcia-Saenz, Isabel Blancas, Isabel Echavarria, Carmen Hinojo, Mireia Margeli, Fernando Moreno, Sonia Pernas, Teresa Ramon y Cajal, Nuria Ribelles, Meritxell Bellet

**Affiliations:** 1 Medical Oncology Department, Hospital Clínico San Carlos, Instituto de Investigación Sanitaria San Carlos (IdISSC), CIBERONC, Madrid, Spain; 2 Hospital Universitario San Cecilio, Instituto de Investigación Biosanitaria de Granada (Ibs.Granada) and Medicine Departmen, Granada University, Granada, Spain; 3 Hospital General Universitario Gregorio Marañón, Instituto de Investigación Sanitaria Gregorio Marañon (IiSGM), CIBERONC, Madrid, Spain; 4 Hospital Universitario Marqués de Valdecilla, Santander, Spain; 5 Institut Català d’Oncologia (ICO)-Badalona (Hospital Germans Trias i Pujol), B-ARGO (Badalona Applied Research Group in Oncology) and CARE (Translational Program in Cancer Research), Badalona, Spain; 6 Institut Català d’Oncologia (ICO)-L’Hospitalet, Institut d’Investigacio Biomedica de Bellvitge (IDIBELL), L’Hospitalet de Llobregat, Barcelona, Spain; 7 Hospital de la Santa Creu i Sant Pau, Barcelona, Spain; 8 UGCI Oncología Intercentros, Hospitales Universitarios Regional y Virgen de la Victoria (IBIMA), Málaga, Spain; 9 Hospital Universitario Vall D’Hebron, and Vall d’Hebron Institute of Oncology (VHIO), Barcelona, Spain

**Correction to: Clinical and Translational Oncology** 10.1007/s12094-023-03203-8

In the sentence beginning “Therefore, tucatinib–lapatinib–capecitabine…” in this article, the text “tucatinib–lapatinib–capecitabine” should have read “tucatinib–trastuzumab–capecitabine” and the correct sentence should read as follows:

Therefore, tucatinib–trastuzumab–capecitabine may be a good option for patients treated with previous T-DM1 and/or T-Dxd [II, B].

The original article has been corrected.

